# Evaluation of Senegal’s prevention of mother to child transmission of HIV (PMTCT) program data for HIV surveillance

**DOI:** 10.1186/s12879-018-3504-z

**Published:** 2018-11-20

**Authors:** Ousmane Diouf, Astou Gueye-Gaye, Moussa Sarr, Abdou Salam Mbengue, Christopher S. Murrill, Jacob Dee, Papa Ousmane Diaw, Ndeye Fatou Ngom-Faye, Pape Amadou Niang Diallo, Carlos Suarez, Massaer Gueye, Aminata Mboup, Coumba Toure-Kane, Souleymane Mboup

**Affiliations:** 1 Laboratory of Bacteriology and Virology of Aristide Le Dantec University Hospital, Dakar, Senegal; 2 Westat, 1600 Research Blvd, WB 258, Rockville, MD 20850 USA; 3 Centers for Disease Control and Prevention, 1600 Clifton Road, Atlanta, USA; 4 Division pour la Lutte Contre Le SIDA et les ISTs (DLSI), Dakar, Senegal; 5 Conseils National pour la Lutte Contre le SIDA (CNLS), Dakar, Senegal; 6 Present Address: IRESSEF: Institut de Recherche en Santé, de Surveillance Epidémiologique et de Formations, Arrondissement 4 Rue 2 D1, Pole Urbain de Diamniado, 7325 Dakar, BP Senegal

**Keywords:** HIV, Senegal, HIV surveillance, Sentinel surveillance, Prevention of mother-to-child transmission, Antenatal clinic

## Abstract

**Background:**

With the expansion of Prevention of Mother to Child Transmission (PMTCT) services in Senegal, there is growing interest in using PMTCT program data in lieu of conducting unlinked anonymous testing (UAT)-based ANC Sentinel Surveillance. For this reason, an evaluation was conducted in 2011–2012 to identify the gaps that need to be addressed while transitioning to using PMTCT program data for surveillance.

**Methods:**

We conducted analyses to assess HIV prevalence rates and agreements between Sentinel Surveillance and PMTCT HIV test results. Also, a data quality assessment of the PMTCT program registers and data was conducted during the Sentinel Surveillance period (December 2011 to March 2012) and 3 months prior. Finally, we also assessed selection bias, which was the percentage difference from the HIV prevalence among all women enrolled in the antenatal clinic and the HIV prevalence among women who accepted PMTCT HIV testing.

**Results:**

The median site HIV prevalence using routine PMTCT HIV testing data was 1.1% (IQR: 1.0) while the median site prevalence from the UAT HIV Sentinel Surveillance data was at 1.0% (IQR: 1.6). The Positive per cent agreement (PPA) of the PMTCT HIV test results compared to those of the Sentinel Surveillance was 85.1% (95% CI 77.2–90.7%), and the percent-negative agreement (PNA) was 99.9% (95% CI 99.8–99.9%). The overall HIV prevalence according to UAT was the same as that found for women accepting a PMTCT HIV test and those who refused, with percent bias at 0.00%. For several key PMTCT variables, including “HIV test offered” (85.2%), “HIV test acceptance” (78.0%), or “HIV test done” (58.8%), the proportion of records in registers with combined complete and valid data was below the WHO benchmark of 90%.

**Conclusions:**

The PPA of 85.1 was below the WHO benchmarks of 96.6%, while the combined data validity and completeness rates was below the WHO benchmark of 90% for many key PMTCT variables. These results suggested that Senegal will need to reinforce the quality of onsite HIV testing and improve program data collection practices in preparation for using PMTCT data for surveillance purposes.

## Background

Senegal is one of the countries where the HIV epidemic is concentrated, with an HIV prevalence of less than 1% in the general population [1] but very high (up to 30%) among certain key populations such as female sex workers (FSWs) and men who have sex with men (MSM) [2].

The HIV prevalence obtained from sentinel surveillance conducted among pregnant women attending antenatal clinics (0.7%) is comparable to what is seen in the general population (< 1%) [3, 4].

Senegal started conducting HIV sentinel surveillance among pregnant women attending antenatal clinics (ANC) as early as 1989, based on WHO/UNAIDS recommendation [5]. Initially, ANC sentinel surveillance (SS) in the country was limited to only 4 regions, and this was gradually extended to cover all 14 regions of the country by 2004.

By the time we conducted this study in 2011–2012, ANC Sentinel Surveillance was conducted in 45 sites, selected from a total of 198 existing ANCs in the country. Women who attended ANC were generally representative of all pregnant women in Senegal due to high (96%) ANC coverage [3, 4] and uptake (94%) [4].

The ANC Sentinel Surveillance rounds were conducted every two years using the unlinked anonymous HIV testing (UAT) method. The UAT method is defined by the collection of left-over blood from routine syphilis testing that has been stripped of all personal identifiers and that is irreversibly made anonymous [5–7]. The left-over blood is then used for HIV testing during the sentinel surveillance surveys. And, in these situations, individuals whose blood tests are positive for HIV cannot be contacted or informed of their status [5].

The Division for the Control of AIDS and Sexually Transmitted Diseases/Ministry of Health (MOH) started offering rapid HIV tests at ANC clinics as part of Senegal’s national prevention of mother-to-child transmission (PMTCT) program in 2006 [3]. With the expansion of HIV testing in PMTCT program settings in Senegal, all 45 ANC sites engaged in the HIV Sentinel Surveillance program now provide HIV rapid testing to pregnant women attending ANC services.

The current ANC sentinel surveillance based on unlinked anonymous testing (UAT) has been an important data source for monitoring the HIV epidemic, program planning, and response in the country. However, with the expansion of PMTCT services, currently covering 96% of all pregnant women [3, 4], there is growing interest in using PMTCT program data in lieu of continuing UAT-based ANC Sentinel Surveillance [8]. PMTCT programs collect socio-demographic and HIV testing data similar to that collected by ANC, and it has distinct advantages over ANC surveillance data such as: (a) the potential for larger geographic surveillance coverage that can make HIV estimates more representative, and (b) anticipated lower cost of using PMTCT data for surveillance purposes [8]. Additionally, UAT-based ANC HIV surveillance systems raises ethical concerns as pregnant women tested for HIV in the context of surveillance do not receive their surveillance test result; although they are offered routine HIV testing through the PMTCT program.

However, the use of routine data from PMTCT programs to estimate HIV prevalence may be challenged by factors such as: varying quality of individual-level PMTCT program data at the sites, lack or low-quality control (QC)/quality assurance (QA) for PMTCT HIV testing at the sites, or selection bias which may be due to potential associations between acceptance of PMTCT HIV testing and likelihood of being HIV-positive or of known HIV-positive status [8–10].

Therefore, to effectively transition to utilizing programmatic data for surveillance purposes, it is necessary to assess similarity between UAT surveillance and PMTCT program HIV test results, evaluate the quality of routine PMTCT program data and testing, and identify any potential biases that may be introduced by differences in routine testing uptake [8]. We also assessed the quality of routine PMTCT HIV data and testing to determine the challenges that need to be overcome to use programmatic data as the basis for sentinel surveillance among pregnant women attending ANC clinics, in accordance with WHO-guidelines [8, 9]. In 2013, the WHO published guidelines to support country assessments of their readiness to transition from ANC sentinel surveillance to PMTCT-based sero-surveillance [8]. This was followed in 2015 by the Guidelines for Conducting HIV Surveillance among Pregnant Women Attending Antenatal Clinics Based on Routine Program Data [9].

## Methods

The methodology for Sentinel Surveillance in Senegal is based on Joint United Nations Programme on HIV/AIDS (UNAIDS)/World Health Organization (WHO) recommendations for monitoring HIV among pregnant women attending ANC and has been described elsewhere [5, 6]. Data from HIV sentinel surveillance surveys are not only used to monitor HIV trends among pregnant women but are also applied as a proxy to estimate the HIV prevalence and incidence among adults in the general population using tools developed by the Joint United Nations Programme on HIV/AIDS (UNAIDS) [5, 6]. Over the past two decades, data from such surveys have been key in monitoring the control of the HIV epidemic in many countries [7].

Conducting nationwide general population surveys such as Demographic and Health Surveys (DHSs) require significant financial resources for Low Income Countries and are conducted approximately on a 5-year intervals basis, while lower cost than the later surveys such as the sentinel surveillance among pregnant women are recommended by the world health organization (WHO) on a more frequent basis (about every 2 years) [5, 6].

### Study size and sample size

The UAT surveillance round was conducted between December 2011 and March 2012 in 45 sites. All 45 ANC Sentinel Surveillance sites provided PMTCT HIV testing services and were included in the current study. Sites were chosen to represent all 14 regions of Senegal, with two urban sites and at least one rural site per region. As recommended by the WHO guidelines, [5, 8] the 45 sites were selected through convenience sampling based on practicality (physical accessibility; sufficient volume of ANC clients to reach sample-size requirements of 300 and 150 women within a 3-months period for urban and rural sites, respectively); and characteristics that the national surveillance program wanted to be represented in surveillance (urban and rural). Overall, the 45 selected sites included 27 urban and 18 rural sites.

Three hundred women were selected in each urban site and 150 in each rural site for UAT HIV testing during the Sentinel Surveillance period. The inclusion criteria were as follow: any woman with a confirmed pregnancy; Aged between 15 and 49 years, attending first prenatal visit, and providing consent for the PMTCT HIV testing. For the needs of PMTCT data quality assessment and based on WHO recommendations [8], clinic records of 45 pregnant women were selected during the UAT Sentinel Surveillance period and 3 months prior from each site.

### HIV testing, data collection tools and methods

#### UAT surveillance biological specimen

The UAT surveillance round was conducted between December 2011 and March 2012, during which women attending ANC clinics for the first time during the current pregnancy were included for the biological survey at participating sites.

All pregnant women were offered the routine antenatal clinic tests which included syphilis testing. Each woman who consented to syphilis testing had 5-ml of blood drawn. After syphilis testing, left-over blood samples from eligible women were transferred into a separate anticoagulant tube, stripped of all personal identifiers and then sent for HIV testing to the HIV reference laboratory, an ISO 15189 accredited medical laboratory in Dakar, Senegal. The algorithm of the Sentinel Surveillance used a combined 4th generation enzyme-linked immunosorbent assay (ELISA) test using either Murex1.2.0, or Enzygnost Integral. Samples found to be positive at the first ELISA were retested with a rapid test (using either Immuno-Comb or Hexagon HIV1/2) that distinguishes between HIV-1, HIV-2 and HIV 1&2 positive results. Samples with discordant results from the first two tests were confirmed with the Western Blot (WB) test (using either HIV BLOT 2.2 or INO-LIA 1.2 score). All HIV test results were double entered into a database and all data management procedures were conducted at the reference laboratory.

#### PMTCT HIV testing

All pregnant women attending ANC were also offered HIV testing and counseling through the PMTCT services: those who accepted were tested and received their results the same day.

For routine PMTCT program testing in ANC clinics, HIV testing was performed using rapid testing, with first Determine (Alere, Inc., Waltham, Massachusetts, USA) or SD Bioline (Standard Diagnostics, Inc. Yongin-si, South Korea) as an initial screening test at the clinic level (Health Post or Health Center). If the result was nonreactive, the woman was considered HIV-negative; but if the result was reactive, confirmation was done with the Bispot (Orgenics, Yavne, Israel) test at the lab level (Health center only). Women with discrepant results were asked to return for repeat testing 30 days later, with re-sampling done at the sites and retesting at the reference laboratory.

#### UAT surveillance data collection form

A data collection form was used by trained the reference laboratory staff to collect the information needed for analysis at the site level. On this paper form, a unique identification number (ID number) was assigned to all eligible pregnant women attending the antenatal clinics during the UAT survey period, whether they accepted PMTCT HIV testing or not. This ID number was not related to any personal characteristics of the women. Key variables captured in the form included socio-demographic data (age, marital status, residence, and education), obstetrical information (gravidity and parity) and PMTCT related information (HIV test offered, HIV test acceptance, HIV test done, and HIV test result delivered). All the data collected from the PMTCT registers were routinely collected by the clinics. The filled forms were then taken to the reference laboratory for data entry and cleaning prior to analysis. In addition to PMTCT HIV test results, HIV test results from UAT Sentinel Surveillance were also included in the form prior to data entry.

#### PMTCT retrospective data quality assessment

To assess the quality of routine PMTCT data, study staff examined ANC and lab registers. Forty-five records were abstracted from the sentinel surveillance period, and another 45 records were abstracted from the 3 months just prior to Sentinel Surveillance period from each of the 45 sentinel surveillance sites. Every 5th woman was selected from the list of the ANC registers until the required number was met. Key PMTCT program parameters such as the date of consultation, age, gravidity, parity, HIV test offered, testing acceptance, testing performance, test results, and delivery of test results were evaluated for data validity and completeness. A value was considered complete if it was present and legible in the field, and valid if it was within the expected range of values for that field.

### Statistical analyses

Analyses were performed using STATA version 12. The overall and site-specific HIV prevalence rates were estimated as a proportion with 95% confidence intervals. The 95 % confidence intervals (CI) were derived from approximation based on normal distribution. All confidence interval calculations aggregating across sites were based on pooled data and did not account for site level clustering.

To be consistent with WHO recommendations, [8] the median of the site-level HIV prevalence rates and related interquartile ranges (IQR) were also reported.

Positive percent agreement (PPA) and negative percent agreement (NPA) were calculated to assess the level of agreement between the UAT surveillance and routine PMTCT test results. A PPA or a NPA below the benchmarks signified that disagreement of the two testing results exceeded what would have been expected due to statistical variability or the performance characteristics of the two testing algorithms, and was likely to be due to human error in operating ANC HSS or PMTCT HIV tests [5]. For a country such as Senegal with a HIV prevalence close to 1%, and using a 2-test Serial algorithm for HIV diagnosis, the benchmarks recommended by the WHO were 96.6 for the PPA of and 99.9 for the NPA [8].

The prevalence ratio between refusers and non-refusers of PMTCT testing and the percent bias due to refusals that would be introduced if ANC surveillance were based on routine PMTCT tests instead of UAT test results were also calculated. Bias was measured by the parameter “selection bias”, defined as the per cent relative change (positive or negative) from the total HIV prevalence.

(among pregnant women who did and did not receive PMTCT HIV testing) to the observed HIV prevalence (among pregnant women who received PMTCT HIV testing) [8]. A percent bias between − 10 and + 10% at all ANC SS sites was considered sufficiently accurate to use for HIV surveillance purposes [8].

All statistical tests were conducted based on two-tailed alternatives and *p* < 0.05 was considered significant.

An Excel spreadsheet provided by the U.S. Centers for Decease Control and Prevention (CDC) was used in calculating the percentage of missing, invalid, valid, and complete data for each parameter in the antenatal registers.

### Ethical considerations

Approval to conduct the study was received from the Senegal Ministry of Health’s National Ethics Committee and from the Center for Global Health (CGH), U.S. Centers for Disease Control and Prevention (CDC) Atlanta.

All ANC surveillance procedures in Senegal were based on UNAIDS/WHO guidelines [5, 6].

As recommended in the 2003 UNAIDS/WHO *Guidelines for conducting HIV sentinel serosurveys among pregnant women and other groups* [5], informed consent was not needed for unlinked anonymous HIV testing (UAT) *as* the left-over blood from routine syphilis testing were stripped of all personal identifiers that could permit personal identification. The privacy of consenting pregnant women and their data confidentiality were ensured through a permanent delinking process that does not allow HIV test results to be traced back to any personal identifying information [5].

Verbal consent was required prior to HIV testing under the PMTCT national public health program. Verbal consent is obtained overall from all women in-country during routine HIV testing as part of the national PMTCT program, as recommended by Senegal’s Ministry of Health.

HIV testing was offered to pregnant women free of charge, and positive cases were enrolled for PMTCT services according to national guidelines.

As a reminder, no PMTCT data or biological specimens were collected from pregnant women solely for the purpose of surveillance.

## Results

**Fig. 1:**
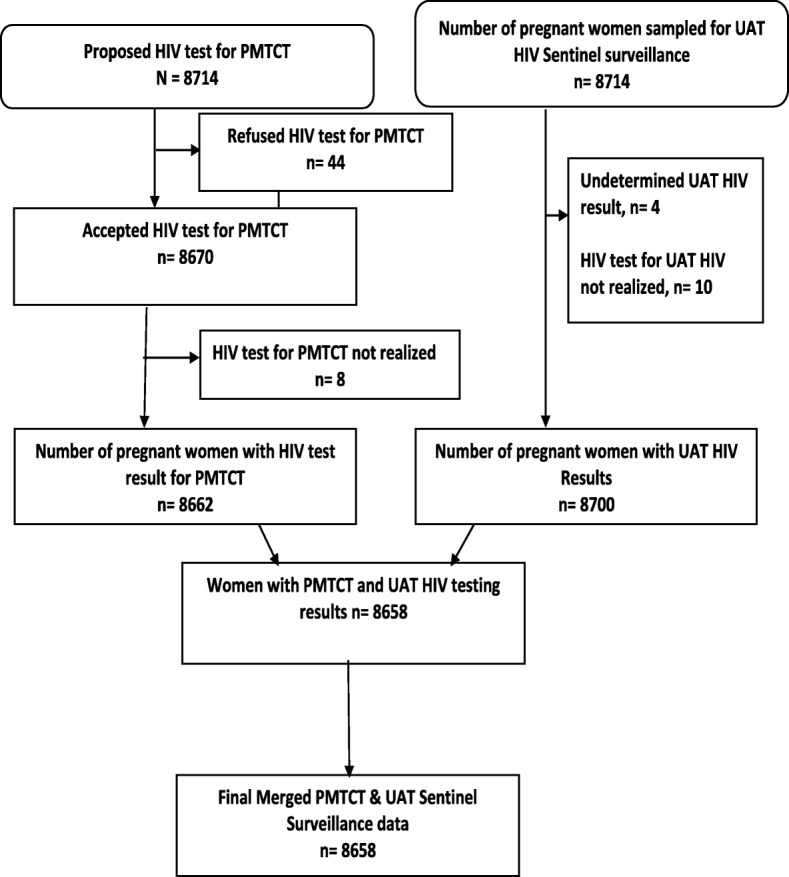
PMTCT program data and UAT surveillance data used

ANC Sentinel Surveillance was conducted in 45 antenatal clinics; including 27 urban sites and 18 rural sites. For the comparison of HIV testing results, 2 sites were excluded because one of the sites was non-functional and the samples from the second site were not appropriately collected and could not be analyzed. Overall, 8714 pregnant women who met the eligibility criteria for unlinked anonymous testing across the remaining 43 sites were initially selected. Of these 8714 pregnant women, 44 (0.5%) were excluded from the comparison because they refused to be tested for HIV as part of the PMTCT program, 8 (0.1%) were excluded because PMTCT HIV testing was not realized, and 14 (0.2%) were excluded because UAT testing was not realized or the results remained indeterminate after WB testing (see Fig. 1). Finally, 8658 pregnant women with both UAT Sentinel Surveillance and routine PMTCT HIV testing results available were included in the final analysis.

For the women included in the analysis, most of them were in the 15–25 years old age range (51.6%), followed by the 26–39 years old group (37.4%), then by the 40+ years old group (2.2%) and finally those who were < 15 years old (0.3%). More than half of the women never went to school (60%) while close to all of the (94.3%) were married. Among those who were married, 24.9% were from a polygamous relationship. The average number of gravidity was 4.4 and the average number parity was 3.2.

The median site HIV prevalence across the 43 sites based on routine PMTCT testing data was 1.1% (Inter Quartile Range or IQR: 1.0) while the median site prevalence calculated from the UAT Sentinel Surveillance data was also at 1.0% (IQR: 1.6) with a non-significant *p*-value. (Table 1) The overall HIV prevalence rate was 1.3% (95% CI: 1.1–1.6) for women from the PMTCT program data and 1.4% (95% CI: 1.2–1.7) for those from the UAT Sentinel Surveillance data (data not shown in table). In absolute terms, the overall HIV prevalence was 0.1% lower when it was measured with the routine PMTCT data versus the Sentinel Surveillance data (*P*-value non-significant).

**Table 1:** Individual-level agreement of PMTCT HIV testing results versus UAT surveillance

Region	Site	N	Comparison between the results of HIV testing for PMTCT (R) and those of Sentinel Surveillance (S)				Positive Percent Agreement	Negative percent agreement	PMTCT HIV Prevalence (%)	UAT surveillance HIV Prevalence (%)
			R-S+	R + S+	R + S-	R-S-				
West	CS Mbao	174	1	4	0	169	80.0	100.0	2.9	2.3
	CS Roi Baudoin	196	0	2	0	194	100.0	100.0	1.0	1.0
	CS de Mbour	300	2	5	2	291	71.4	99.3	2.3	2.3
	CS khombole	272	0	2	0	270	100.0	100.0	0.7	0.7
	Centre de santé 10eme	238	1	7	1	229	87.5	99.6	3.4	3.4
	PMI Medina	300	0	3	0	297	100.0	100.0	1.0	1.0
	PS Nianing	138	0	2	0	136	100.0	100.0	1.4	1.4
	PS Nguekhokh	175	0	0	0	175	NA	100.0	0,0	0,0
	PS de Mboro	175	0	2	0	173	100.0	100.0	1.1	1.1
North	CS Dagana	166	0	1	1	164	100.0	99.4	0.6	1.2
	CS Linguere	99	0	1	0	98	100.0	100.0	1.0	1.0
	CS Matam	219	1	1	0	217	50.0	100.0	0.9	0.5
	CS Louga	272	0	0	0	272	NA	100.0	0,0	0,0
	CS Saint Louis	112	0	0	0	112	NA	100.0	0,0	0,0
	PS Bokidiawé	150	0	0	0	150	NA	100.0	0,0	0,0
	PS Ourossogui	186	0	2	0	184	100.0	100.0	1.1	1.1
	PS de Samyabal	159	0	1	0	158	100.0	100.0	0.6	0.6
Centre	CS Diourbel	297	1	5	0	291	83.3	100.0	2.0	1.7
	CS Fatick	164	0	2	0	162	100.0	100.0	1.2	1.2
	CS Kasnack	299	0	3	0	296	100.0	100.0	1.0	1.0
	CS Koungheul	279	0	2	0	277	100.0	100.0	0.7	0.7
	CS Ndoffane	175	0	3	0	172	100.0	100.0	1.7	1.7
	CS Sokone	293	2	2	0	289	50.0	100.0	1.4	0.7
	CS Touba	298	0	2	0	296	100.0	100.0	0.7	0.7
	CS kaffrine	184	0	3	0	181	100.0	100.0	1.6	1.6
	PS Dianké	119	0	0	0	119	NA	100.0	0,0	0,0
	PS Tataguine	175	0	0	0	175	NA	100.0	0,0	0,0
	PS de Ngoye	163	0	2	0	161	100.0	100.0	1.2	1.2
	SOS Kaolack	300	0	3	0	297	100.0	100.0	1.0	1.0
South	C.S Vélingara	81	0	1	3	77	100.0	96.3	1.2	4.9
	C.S de Goudomp	299	0	4	0	295	100.0	100.0	1.3	1.3
	C.S de Sédhiou	115	0	4	0	111	100.0	100.0	3.5	3.5
	CS C Senghor Ziguinchor	292	1	11	0	280	91.7	100.0	4.1	3.8
	CS Bignona	298	0	9	0	289	100.0	100.0	3.0	3.0
	CS Boumkiling	159	0	2	0	157	100.0	100.0	1.3	1.3
	CS Kolda	298	5	8	0	285	61.5	100.0	4.4	2.7
	CS Salémata	71	0	0	0	71	NA	100.0	0,0	0,0
	CS Saraya	33	0	0	0	33	NA	100.0	0,0	0,0
	CS Tambacounda	301	0	0	0	301	NA	100.0	0,0	0,0
	PS Missira	160	0	0	0	160	NA	100.0	0,0	0,0
	PS Diawara	175	0	0	2	173	NA	98.9	1,1	0,0
	PS Diaobé	150	4	2	0	144	33.3	100.0	4.0	1.3
	PS de Kabrousse	149	0	2	0	147	100.0	100.0	1.3	1.3
Overall		8658	18	103	9	8528	85.1 (77.2–90.7%),	99.9 (95%CI: 99.8–99.9)	Median = 1.1 (IQR: 1.0)	Median = 1.0 (IQR: 1.6)
									*p*-value = non-significant	

1. Percentage of positive agreement = (R + S+/R + S+ and R-S+)

2. Percentage of negative agreement = (R-S-)/ (R-S- and R + S-)

With R−/R+ the results of HIV testing for PMTCT

And S-/S+ the results of HIV Sentinel Surveillance

a) The positive percentage agreement (PPA) was calculated as the proportion of pregnant women tested HIV-positive through ANC SS HIV testing (using a combined 4th generation ELISA) who were identified as HIV-positive through routine PMTCT testing (using a 2-rapid testing serial algorithm)

b) The negative percentage agreement (NPA) was calculated as the proportion of pregnant women tested HIV-negative through ANC SS HIV testing (using a combined 4th generation ELISA) who were identified as HIV-negative through routine PMTCT testing (using a 2-rapid testing serial algorithm)

The overall PPA of PMTCT and UAT Sentinel Surveillance HIV testing was at 85.1% (95% CI: 77.2–90.7), while the overall NPA was 99.9% (95% CI: 99.8–99.9) (data not shown in table). The analysis by region showed an overall PPA of 87.1% (95% CI: 69.2–95.8) in the West, 81.1% (95% CI: 67.6–90.1) in the South, 85.7% (95% CI: 42.0–99.2) in the North and 90.0% (95% CI: 72.3–97.4) in the central regions of the country.

Our results also showed that the proportion of women who accepted PMTCT HIV testing was 99.4% (8658/8714). Among the 44 women who refused the HIV test for PMTCT, we were able to receive blood samples from 42 of them, and among those, one person was diagnosed positive for HIV at the reference laboratory from UAT. HIV prevalence among those who refused the HIV test for PMTCT was 2.3% (95% CI: 0.1–14.1), while among the non-refusers this prevalence was 1.4 (95% CI: 1.2–1.7). The overall HIV prevalence among all women, including those who accepted or refused a PMTCT HIV test, was 1.4% (95% CI: 1.2–1.6), with a low percent bias at 0.00%. (Table 2).

**Table 2:** Effect of missing routine PMTCT HIV data on estimated HIV prevalence rates

ANC Surveillance HIV Prevalence by Routine PMTCT HIV Test Acceptance			
	Total	Refused PMTCT HIV testing	Accepted PMTCT HIV testing
HIV+	122	1	121
Total	8700	42	8658
HIV Prevalence	1.4% (1.2–1.6)	2.3% (0.1–14.1)	1.4% (1.2–1.7)
Measures of Effect of PMTCT Uptake on HIV Prevalence			
Absolute difference^a^	+ 0.9%		
Relative difference^b^	+ 64.3%		
Percent bias^c^	0.00%		

^a^Absolute difference is the HIV prevalence in those whose PMTCT HIV status is missing minus the HIV prevalence in those for whom it is present

^b^Relative difference is the absolute difference divided by the HIV prevalence among those for whom the PMTCT HIV status is present

^c^Percent bias is the HIV prevalence in those for whom PMTCT HIV status is present minus the overall HIV prevalence, divided by the overall HIV prevalence

The results from the quality assessment of routine PMTCT data are shown in Table 3. For this effort, 1 non-functional site was excluded, and the analysis was done using data from 44 sites. The results showed that variables capturing socio-demographic and obstetrical information had very high combined validity and completeness rates. The combined validity and completeness rates were at 98.4% for age, 99.0% for gravidity and 98.2% for parity. However, variables related to PMTCT HIV testing had much lower rates; with combined validity and completeness rates at 85.2% for HIV test offered, 78.0% for HIV test acceptance, 58.8% for test done, and 47.1% for HIV test results delivered. Citing confidentiality issues, we also noticed a limited recording of HIV test results in the ANC registers, with non-standard ways of keeping HIV test results. For example, some mid-wives kept the list of HIV positive test results with them/in their own purse, and some others chose to make red dots in the register in front of those who were positive.

**Table 3:** Quality of data in the antenatal registers

PMTCT program parameters	Number^a^ and Percentage of complete & valid data		Number^b^ and Percentage of sites with less than 90% of complete & valid data	
	*n* = 3960	Mean (%)	*n* = 44	%
Date of visit	3948	99.7	0	0.0
Age	3897	98.4	1	2.3
Number of births	3920	99.0	0	0.0
Number of live births	3889	98.2	2	4.5
PMTCT HIV test offered	3374	85.2	12	27.3
PMTCT HIV test acceptance	3089	78.0	24	54.5
PMTCT HIV test done	2328	58.8	37	84.1
PMTCT HIV test results delivered	1865	47.1	41	93.2

^a^Calculated on 90 pregnant women selected for each site from the register before (*n* = 45 pregnant women) and during (n = 45 pregnant women) the Sentinel Surveillance period. Total = 44 sites participating in DQA × 90 records = 3960 records

^b^Number of PMTCT sites participating in DQA

## Discussion

The HIV prevalence estimate obtained from UAT sentinel surveillance data (1.4%; 95% CI 1.2–1.7) was very similar to the estimate obtained from the PMTCT data (1.3%; 95% CI 1.1–1.6). However, the overall PPA of 85.1% was low compared to the general benchmark of 96.6% recommended by the WHO for a country like Senegal [5]. Assuming that the UAT testing results were accurate, as these were performed at the ISO 15189 accredited national reference laboratory and are considered as the in-country gold standard for HIV testing, the PPA of 85.1% means approximately 15 out of every 100 HIV positive women received an HIV negative result from the PMTCT program. Our findings of low PPA between PMTCT and UAT HIV testing data are similar to what is seen in most similar studies in Africa, with PPAs of 88.5% in Mozambique, 75.9% in Kenya, and 91.2% in Zimbabwe [11–13].

Regarding the data quality assessment results, the combined data completeness and validity of PMTCT data was high for variables capturing socio-demographic and obstetrical information such as age, gestation or gravidity, but was below the WHO benchmark of 90% for many variables capturing key PMTCT program information such as HIV testing uptake or the recording of HIV test results. Citing confidentiality issues, we noticed a limited recording in the ANC registers, with non-standard ways of keeping HIV test results. Similar issues were also seen in Kenya by Seguy et al. [14] who described problems with accessing registers, interpreting nurse handwriting and a lack of standardization between practitioners. Incomplete data on HIV testing uptake and lack of key surveillance variables (such as HIV test results) in ANC registers were also seen and described in Kenya and Uganda [14, 15]. On the other hand, some countries such as Botswana have reported high level of completeness of routine data in ANC surveillance sites [16].

The low overall PPA and low to moderate combined data completeness and validity for key PMTCT variables suggested that Senegal still face gaps in the quality of routine PMTCT data systems, HIV testing, and routine QA for HIV testing [11, 12, 14, 17]. As recommended by the CDC and the WHO, a quality monitoring and strengthening approach will be needed to allow the use of routine PMTCT data for surveillance while providing the opportunity for Senegal to improve its routine clinical, laboratory and surveillance activities by monitoring and strengthening the quality of routine data, routine HIV testing and routine HIV testing quality assurance [18–20].

The work to improve routine HIV testing and data will be done through a combination of training, mentorship, supervision and continuous quality control (QC)/quality assurance (QA) activities [18, 20, 21]. Developing a robust and high-quality routine HIV testing and data system will only be possible through collaborative work and a joint funding effort, bringing together local and international partners such as the Ministry of Health/the national PMTCT program, the national reference laboratory, the US CDC and/or the Global Fund [21, 22]. Finally, there will be a plan to fully integrate all of this effort into the Ministry of Health’s national strategies and plans to guarantee its long-term sustainability [22].

As recommended by the U.S. CDC and the WHO this will be done within a context of a prospective sentinel surveillance design to provide real-time QC/QA to address gaps in routine data and HIV testing and consistently improve HIV data and quality testing standards across all ANC in-country sites [18, 20].

This assessment also found a PMTCT HIV testing uptake of 99.4% and an overall selection bias in the PMTCT program data of 0.00%. This finding means that non-acceptance of HIV testing among pregnant women in antenatal clinics did not introduce a significant bias in the HIV prevalence estimates based on PMTCT testing. When uptake of PMTCT testing is high, the percent bias is constrained. This finding is similar to what is seen in other countries in Africa with high rates of uptake of PMTCT HIV testing among ANC attendees [11, 13].

## Conclusions

Our results showed no statistically significant difference between overall and median HIV prevalence from routine PMTCT data versus HIV prevalence from traditional ANC Sentinel Surveillance data. However, the low overall PPA and the low combined data completeness and validity for some key PMTCT variables suggest that Senegal should reinforce the quality of onsite HIV rapid testing and program data collection practices as it transitions to using PMTCT data for HIV surveillance needs as recommended by the WHO and the CDC.

A combination of training, mentorship, supervision and continuous quality control (QC)/quality assurance (QA) activities to improve the quality of routine PMTCT data and HIV testing will be required as Senegal transitions to the use of PMTCT program data for surveillance needs. This effort to develop a robust and high-quality HIV testing and routine data system will involve all key national and international partners, and will be integrated into the Ministry of Health’s national strategies and plans to guarantee its long-term sustainability.

A prospective sentinel surveillance design is recommended in this setting to provide real-time quality control to address gaps in routine data and HIV testing to allow the use of routine PMTCT data for surveillance while also providing an opportunity for Senegal to improve its routine PMTCT program data and related clinical and laboratory activities in ANC Clinics.

## Abbreviations

AIDS: Acquired Immunodeficiency Syndrome
ANC: Antenatal Clinics
CDC: U.S. Centers for Decease Control and Prevention
CGH: Center for Global Health
ELISA: Enzyme-linked Immunosorbent Assay
FSW: Female Sex Workers
HIV: Human Immunodeficiency Virus
IQR: Interquartile Range
MOH: Ministry of Health
MSM: Men who have Sex with Men
NPA: Negative Percent Agreement
PMTCT: Prevention of Mother to Child Transmission
PPA: Positive Percent Agreement
QA: Quality Assurance
SS: Sentinel Surveillance
UAT: Unlinked Anonymous Testing
WB: Western Blot
